# Increases in arm volume predict lymphoedema and quality of life deficits after axillary surgery: a prospective cohort study

**DOI:** 10.1038/s41416-020-0844-4

**Published:** 2020-05-04

**Authors:** Nigel Bundred, Phil Foden, Chris Todd, Julie Morris, Donna Watterson, Arnie Purushotham, Maria Bramley, Katie Riches, Tracey Hodgkiss, Abigail Evans, Anthony Skene, Vaughan Keeley, the Investigators of BEA/PLACE studies

**Affiliations:** 1 Departments of Surgery and Medical Statistics, Manchester University NHS Foundation Trust, Manchester, UK; 2 Manchester Academic Health Sciences Centre (MAHSC), Manchester, UK; 3 Faculty of Biology, Medicine and Health, School of Health Sciences, The University of Manchester, Manchester, UK; 4 Guyʼs & St Thomas’ NHS Foundation Trust, London, UK; 5 Pennine Acute Hospitals NHS Trust, Greater Manchester, UK; 6 University Hospitals of Derby and Burton NHS Foundation Trust, Derby, UK; 7 Poole NHS Foundation Trust, Dorset, UK; 8 Royal Bournemouth NHS Foundation Trust, Bournemouth, UK; 9 University of Nottingham Medical School, Nottingham, UK

**Keywords:** Quality of life, Breast cancer

## Abstract

**Background:**

Lymphoedema develops after axillary clearance (ANC) in 25% of patients. This prospective, multi-centre study compared multi-frequency bioimpedance spectroscopy (BIS) with arm volume measurement to: (1) determine which test has better diagnostic accuracy, (2) identify factors predicting development of lymphoedema, and its effect on quality-of-life.

**Methods:**

Participants (*N* = 1100) underwent measurements pre and post-ANC surgery for breast cancer. Relative arm volume increase (RAVI) of >10% diagnosed lymphoedema. Predictors of lymphoedema were determined using logistic regression. Optimal diagnostic method was assessed using diagnostic accuracy. Quality-of-life was assessed using the FACT B + 4 questionnaire.

**Results:**

Lymphoedema was diagnosed in 22.8% women using RAVI > 10%, 45.6% using BIS criteria, while 24.5% underwent compression sleeve application by 24 months. BMI > 30 was an independent factor for both development (*p* = 0.005) and progression (*p* = 0.015) of lymphoedema. RAVI at 1 month, BMI > 30 and number of involved nodes contributed to a novel scoring model to predict lymphoedema by 36 months. Larger decreases in QoL scores post-surgery occurred in lymphoedema patients (*p* < 0.001). Progression to moderate lymphoedema occurred in 15% patients after sleeve application.

**Conclusions:**

RAVI measurement was the best diagnostic tool for lymphoedema. BIS alone is not appropriate for lymphoedema screening or diagnosis. BMI > 30 predicted lymphoedema diagnosis and progression.

## Introduction

Lymphoedema, (swelling of the arm after surgery to the axilla), causes arm pain, heaviness, swelling and psycho-social morbidity in cancer patients.^1–5^ Lymphoedema increases risk of infections (cellulitis), and increases fears of cancer recurrence.^2,3^ Although recent trials suggest clinically node negative breast cancers found at surgery with two or fewer positive nodes can avoid further axillary surgery,^2^ clinically node positive patients (Stage 2/3) continue to receive axillary clearance (ANC) or radiotherapy after sentinel node biopsy. Both of these predispose to lymphoedema development.^2,3^ Whilst prevention of cancer recurrence is the priority for treatment, it is important to identify which patients are at risk of complications is of considerable importance in order to target ancillary management strategies to prevent those complications.

Although screening for lymphoedema is recommended,^6^ the evidence base for diagnosis is limited. There is currently no internationally agreed gold standard definition of what constitutes lymphoedema following ANC.^1,7–10^ The incidence of lymphoedema varies according to the definition used, ranging from 21.5% in studies using objective measures to over 50% when using patient reported symptoms.^1,4,7–10^

Proposed definitions for lymphoedema include: (a) 200 ml limb volume difference^1,4,7–9^ (b) 10% relative arm volume increase (RAVI)^1,2,9^ (c) 2.0 cm circumferential difference at any point between arms^7,8^ and (d) l-Dex bioimpedance increase of either 7.5 (2sd) or 10 units or greater.^11–13^ Although widely accepted as diagnostic criteria, these different definitions are not equivalent.^7,8^ External compression-sleeves are used as treatment for lymphedema but the threshold criterion for identifying patients benefiting from their use is not evidence based.^7^

Perometry is a standardised infrared optoelectronic technology used to detect and quantify limb volume changes.^14–18^ In early stages of lymphoedema development, the swelling is believed to be due mainly to extracellular fluid accumulation (often considered ‘subclinical’). Multi-frequency bioimpedance spectroscopy (BIS) is a non-invasive technique to measure extracellular fluid, which involves passing an extremely small alternating electric current through the body and measuring the impedance (or resistance) to the flow of this current.^11–13^ BIS compares quantitatively the amount of extracellular fluid in the ‘at risk’ limb (i.e. operated side) with the un-operated side. A three-standard deviation (sd) increase in BIS is the criterion to diagnose lymphoedema (l-Dex>10. Impedimed) although a 2sd deviation (l-Dex>7.5) is also used.^12,13^

A small study suggested BIS detects lymphoedema development up to 10 months before arm volume changes with a sensitivity of 98% and a specificity of 100%,^11^ but subsequent studies have found limited correlation with lymphoedema development after surgery.^12^ BIS is used in American centres for early detection of lymphoedema after surgery to inform treatment in women after axillary surgery.^12,13^

Conventional advice is arm swelling at 3 months post-surgery does not portend chronic swelling and is treated conservatively.^7,8^ Arm measurements are not routine practice in Europe prior to ANC, but prospective studies have demonstrated that RAVI of ≥5% identifies increased risk of subsequent lymphoedema and is a suitable threshold for considering early intervention.^16,17^

Several American guidelines (National Lymphoedema Network Guidelines^10^) recommend screening for lymphoedema.^10^ However, the evidence base for diagnosis (and screening) is limited.^7–10,17^ An observational study of 43 women developing RAVI > 3% after surgery, and prescribed graduated compression sleeves for a duration of 4.4 weeks, observed arm volume reductions of 4.1% using the sleeve.^17^ This reduction was maintained over an average follow-up period of 4.8 months after discontinuing the intervention. Despite limited follow-up and intervention periods, this small study has been cited to argue for pre-operative baseline measurements and led internationally to lymphoedema guidelines recommending early intervention in subclinical lymphoedema with compression garments to prevent the subsequent development of clinical lymphoedema. The optimal threshold and diagnostic tool, for intervention to prevent progression to lymphoedema however remains unclear.

Post-operative surveillance is also advocated to detect early clinical lymphoedema so that early intervention to reduce the risk of progression and complications such as cellulitis can be carried out. To facilitate this a standardised objective method of diagnosing early lymphoedema is required.

In this prospective multicentre study, we sought to (1) compare five methods for diagnosis of lymphoedema, (2) identify which patients were at risk of developing lymphoedema, (3) develop a criterion score to identify patients who would benefit from compression sleeve application and (4) determine the relationship between lymphoedema diagnosis and quality-of-life.

## Patients and methods

In a multicentre prospective study, women undergoing ANC for breast cancer from nine UK centres between July 2010 and May 2014 received pre-operative baseline arm measurements using perometry (RAVI, absolute arm circumference and volume changes) and BIS, then underwent follow-up measurements with both measurement techniques for 5 years.^16^

The primary aim was to determine whether BIS (l-Dex) had equal accuracy (sensitivity and specificity) as perometry for the detection of lymphoedema. Secondary aims were to determine the effect of diagnosis on quality-of-life, cancer survival and identify predictors of lymphoedema. The National Research Ethics Service Committee approved the study and informed consent was required for study participation.

The protocol (see supplementary data), predefined lymphoedema as a RAVI as equal to or >10% relative arm volume increase upper arm or lower arm or both from baseline (compared to the contralateral arm). BIS criterion for diagnosis is a two or three standard deviation (sd) increase in BIS (l-Dex increase >7.5(2sd) or >10(3sd). Impedimed)^12,13^ from baseline.

A mean of two arm measurements at each visit using a 350S Perometer with standard software (Pero System, Germany) reduced intra-observer variability. RAVI was calculated by a formula (see BEA Protocol in appendix) $( {\frac{{A2 \, - \, U2}}{{U2}}} )\times\;100 - ( {\frac{{A1 \, - \, U1}}{{U1}}} )\times\;100$, which allows for changes in the contralateral limb reducing the influence of arm dominance.^14^ Perometry provides arm volume and circumference measurements. Extracellular fluid was measured using the l-DEX®U400 bioimpedance spectroscopy device from ImpediMed Ltd., Australia. Participants who developed lymphoedema (defined by RAVI > 10%) were prescribed a circular-knit compression-sleeve garment.

In cases when swelling of the lower arm, hand or symptoms (but RAVI < 10%) led to compression-sleeve application 20–25 mmHg (sleeves applied outside protocol indications) perometer readings and notes for patients with ipsilateral sleeve applied were reviewed. If the forearm had a RAVI > 10% increase, or if notes indicated hand oedema resulted in sleeve application, sleeve application was used as a clinical surrogate marker of lymphoedema. Time to lymphoedema (RAVI > 10% definition) are presented. RAVI and BIS changes were compared to determine, sensitivity and specificity, in lymphoedema diagnosis.

### Self-reported symptoms

Patients completed the Lymphoedema and Breast Cancer Questionnaire (LBCQ), which has been shown to predict lymphoedema,^17^ and the Functional Assessment of Cancer Therapy Breast+4 (FACT-B + 4)^5^ arm morbidity quality-of-life questionnaire^5^ (in which patients rate arm symptoms from 0 to 5) pre-operatively and at 3, 6, 12 months, and then annually to 60 months.^17^ FACT-B + 4 provides several summary scores^5^ including: the FACT-B Total Score and Trial Outcome Index (TOI). TOI score changes of 5 or more are clinically relevant.^5^

Statistical analysis included sensitivity and specificity analyses of the BIS L-Dex score against the ‘gold standard’ of RAVI assessment post-surgery. Independent samples *t*-tests, Mann–Whitney U tests and chi-squared tests were used to assess the difference between those who did and those who did not develop lymphoedema. Variables with a highly skewed distribution were transformed to obtain an approximate normal distribution. Generalised estimating equations (GEEs) were used to assess quality-of-life changes over time in relation to sleeve application. Logistic regression analysis was used to identify the predictors of lymphoedema by 24 months and develop a scoring model. Cox proportional hazards regression models were used for time to event analyses. Multiple testing was accounted for by using a 1% significance level for univariate analyses and a 5% significance level in the multivariable analyses. All statistical analyses were performed in SPSS 22.

The sample size of 1100 allowed detection of sensitivity and specificity differences between BEA and RAVI (perometer) measurement of 5% or less.

## Results

**Fig. 1:**
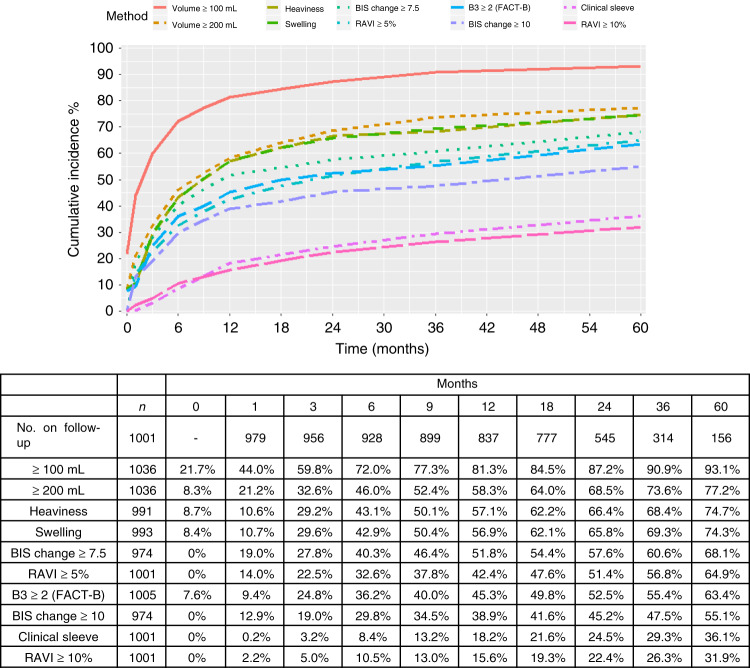
Changes in self-reported symptoms from ARM subscale and objective measures of arm swelling compared to Lymphoedema sleeve application. Frequency of self-reported and objective symptoms of lymphoedema compared to Lymphoedema sleeve application. Diagnosis of Lymphoedema based on RAVI > 10%. Frequency of objective and self-reported definitions of Lymphoedema by time. The B3 question is part of the Fact-B + 4 self-reported questionnaire and the heaviness and swelling are from the lymphoedema checklist questionnaire. *N* represents the number assessed for each modality at baseline and the numbers at each timepoint.

The incidence of lymphoedema defined by RAVI > 10% by 24 months was 22.4%, compared with 45.2% using BIS l-Dex≥10 criterion (or 57.6% using BIS l-Dex >7.5; [2sd]), but only 24.5% of patients required external compression sleeve treatment (Fig. 1) in the 1100 study patients (median follow-up 36.0 months, IQR 23.8–48.3). The extra sleeve application compared to RAVI > 10% was for hand oedema or smaller arm volume changes.

**Fig. 2:**
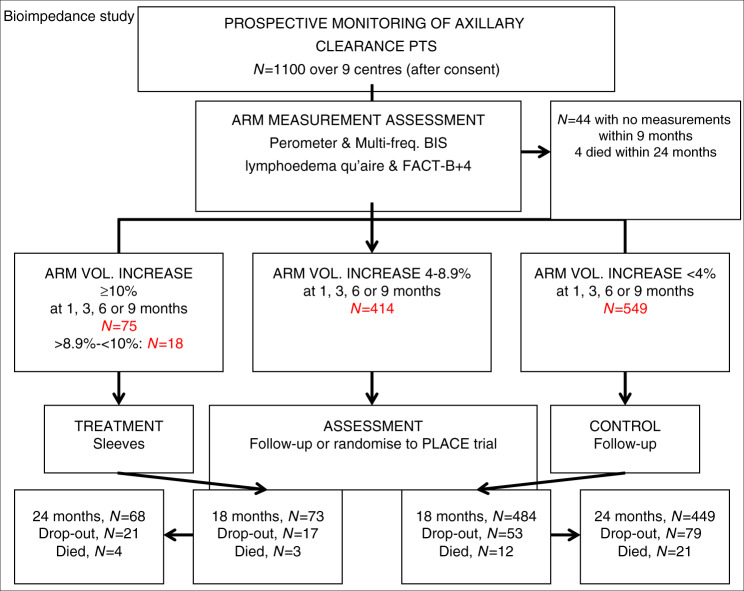
CONSORT diagram showing recruitment to BEA study and numbers with arm volume increase >10%: 4–9% or less than 4% over the first 9 months of the study. Bottom boxes show patients lost to followup, or dying.

Median time to developing lymphoedema was 11.3 months (IQR 5.9–23.8: Table 1). Overall 309 patients withdrew from the study, were lost to follow-up or died (*N* = 105; Fig. 2). Baseline measurements found 8.3% of patients exhibited marked volume (≥200 ml) differences between arms (Fig. 1).

**Table 1:** Age and clinicopathological variables of BEA patients.

Variable		Full cohort	Lymphoedema—RAVI ≥ 10% by 24 months (*N* = 194)	No lymphoedema—RAVI < 10% by 24 months (*N* = 807)
Baseline variables				
Age—Mean (SD) [range]		55.7 (12.4) [22–90]	57.5 (11.7) [28–86]	55.4 (12.5) [22–90]
BMI (pre-op)—Median (IQR) [range]		27.3 (24.0–31.2) [16.6–60.0]	28.3 (24.7–33.3) [17.2–48.2]**	26.9 (23.5–30.6) [16.6–55.6]**
≤25		352 (32.9%)	52 (27.2%)	282 (35.8%)
>25–≤30		385 (35.9%)	62 (32.5%)	281 (35.7%)
>30		334 (31.2%)	77 (40.3%)	224 (28.5%)
Side of ANC and dominant hand Same: Different		564 (51.5%): 532 (48.5%)	91 (46.9%): 103 (53.1%)	427 (52.9%): 380 (47.1%)
Smoker-Never		651 (59.5%)	116 (60.1%)	486 (60.3%)
Ex-smoker		319 (29.2%)	58 (30.1%)	230 (28.5%)
Current smoker		124 (11.3%)	19 (9.8%)	90 (11.2%)
Previous SN biopsy:Yes		368 (34.3%)	55 (28.5%)	289 (36.1%)
Type of surgery				
ANC		258 (23.7%)	41 (21.1%)	197 (24.4%)
WLE + ANC		318 (29.2%)	58 (29.9%)	236 (29.2%)
Mastectomy + ANC		513 (47.1%)	95 (49.0%)	374 (46.3%)
Histology				
Inf ductal		771 (70.9%)	135 (69.6%)	570 (70.8%)
Inf lobular		125 (11.5%)	16 (8.2%)	96 (11.9%)
Mixed invasive		91 (8.4%)	22 (11.3%)	65 (8.1%)
Other		100 (9.2%)	21 (10.8%)	74 (9.2%)
Pathological tumour size, mm Median (IQR) [range]		26.0 (18.0–40.0) [0–220]	29.0 (20.0–43.0) [0–220]	25.0 (17.0–39.8) [0–180]
Grade 0/1		70 (6.5%)	9 (4.7%)	59 (7.4%)
Grade 2		477 (44.2%)	76 (39.6%)	362 (45.2%)
Grade 3		501 (46.4%)	97 (50.5%)	362 (45.2%)
Ungraded		32 (3.0%)	10 (5.2%)	18 (2.2%)
Number of nodes	Removed	17.0 (13.0–23.0) [1–56]	18.0 (14.0–24.0) [2–51]	17.0 (13.0–23.0) [1–49]
Median (IQR) [range]	Involved	2.0 (1.0–5.8) [0–46]	3.0 (1.0–9.0) [0–39]**	2.0 (1.0–5.0) [0–46]**
Node Positive		985 (90.5%)	179 (92.3%)	726 (90.1%)
ER Negative: ER Positive		208 (19.4%): 864 (80.6%)	47 (25.0%): 141 (75.0%)	143 (18.0%): 653 (82.0%)
HER-2	Negative Amplified: 3+	811 (75.7%) 82 (7.6%) :179 (16.7%)	133 (70.0%) 20 (10.5%): 37 (19.5%)	610 (76.8%) 55 (6.9%): 129 (16.2%)
ER negative, HER-2 negative		137 (12.9%)	26 (13.9%)	96 (12.1%)
ER negative, HER-2 amplified/3+		71 (6.7%)	21 (11.2%)	47 (5.9%)
ER positive, HER-2 negative		671 (62.9%)	106 (56.7%)	512 (64.7%)
ER positive, HER-2 amplified/3+		187 (17.5%)	34 (18.2%)	136 (17.2%)
Post-op Radiotherapy		878 (82.7%)	168 (87.5%)	644 (81.0%)
Post-op Chemotherapy		713 (67.3%)	135 (70.3%)	523 (66.0%)
Post-op Endocrine Therapy		874 (82.4%)	151 (78.6%)	663 (83.5%)
Outcome (follow-up) variables				
Recurrence		19 (1.7%)	5 (2.6%)	13 (1.6%)
local/regional only: distant		112 (10.2%)	31 (16.0%)*	70 (8.7%)*
Time to distant recurrence, months Median (IQR) [range]		17.3 (9.1–31.2) [0.6–60.5]	21.2 (11.4–37.0) [0.6–60.5]	16.2 (8.4–29.7) [0.9–54.6]
Time in study, months Median (IQR) [range]		36.0 (23.8–48.3) [0.7^a^–66.2]	36.1 (24.0–48.6) [4.8–63.2]	35.9 (23.7–48.2) [0.7–66.2]

^a^There were 17 patients who withdrew (or lost to follow-up) from the study prior to (or on the date of) definitive surgery and have negative values when using the surgery date as the start point.

Due to multiple testing, only *p*-values < 0.01 were counted as significant in the above table.

*P < 0.01 between the lymphoedema (RAVI ≥ 10%) and no lymphoedema groups.

**P < 0.001 between the lymphoedema and no lymphoedema groups.

For BIS > 10 and >7.5 at 6 months the sensitivity for lymphoedema was 69% (95% CI: 60–84%) and 80% (95% CI: 69–88%) and specificity of 82% (95% CI: 80–84%) and 77% (95% CI: 74-80%) with a PPV of 28% (95% CI: 23–33%) and 24% (95% CI: 19–30%). l-Dex sensitivity and specificity for lymphoedema were unaltered at 24 months 68%, (95% CI: 60–75%), and 79%, (95% CI: 77–82%) respectively), although the PPV was improved at 31% (95% CI: 26–37%) (Table 2).

**Table 2:** Sensitivity and specificity of BIS by 6 and 24 months for lymphoedema diagnosis (RAVI > 10% and sleeve application).

	No lymphoedema (RAVI < 10%)	Lymphoedema (RAVI > 10%)	Total number	No clinical sleeve	Clinical sleeve	Total number
By 6 months						
BIS (<10)	698 (82%) Specificity	27 (31%) PPV	725	690 (80%)	34 (46%)	724
BIS (≥10)	153 (18%) NPV	59 (69%) Sensitivity	212	170 (20%)	40 (54%)	210
RAVI < 10%	–	–	–	820 (94%)	45 (60%)	865
RAVI > 10%	–	–	–	55 (6%)	30 (40%)	85
After 6 months up to 24 months						
BIS ( < 10)	572 (79%)	32 (32%)	604	556 (80%)	65 (49%)	621
BIS ( ≥ 10)	150 (21%)	68 (68%)	218	136 (20%)	69 (51%)	205
RAVI < 10%	–	–	–	667 (94%)	86 (61%)	753
RAVI > 10%	–	–	–	39 (6%)	55 (39%)	94

BIS > 10 increase at 24 months l-Dex sensitivity (68%, 95% CI: 60–75%), and specificity 79%, 95% CI: 77–82% for lymphoedema at 24 months PPV (31%, 95% CI:26–37%) compared to RAVI > 10%

There was a moderate correlation between RAVI and BIS L-Dex at 6 months (*r* = 0.62). BIS diagnostic accuracy was 89.3% and 85.7% at 6 and 24 months respectively (Table 2). Sensitivity and specificity for BIS (l-Dex) fell below the 95% required in the protocol.

Symptoms (swelling (*p* < 0.001), heaviness (*p* = 0.001)) at 24 months were associated with larger increases (from 6 to 24 months) in the exact RAVI but not exact BIS values (Table S1).

Arm swelling category by 6 months predicted lymphoedema by 24 months (*p* < 0.001). Women who developed a RAVI of ≥5 to <10% developed lymphoedema in 29% of cases, whereas a RAVI < 3% by 6 months had a 6% lymphoedema rate by 24 months (Table 3). BIS 5-10 increase by 6 months did not predict lymphoedema at 24 months (Table 3).

**Table 3:** Comparison of Multifrequency Bioimpedance (BIS) and RAVI values by 6 months against subsequent lymphoedema by 24 months.

Multifrequency Bioimpedance (BIS)						
BIS value by 6 months	Lymphoedema defined by RAVI > 10%		Clinical sleeve application		*P*-value	Hazard Ratio
	No lymphoedema (*N* = 737)	Lymphoedema (*N* = 99)	No lymphoedema (*N* = 707)	Lymphoedema (*N* = 135)		
<3	348 (90%)	38 (10%)	345 (91%)	33 (9%)	0.26^a^	1
≥3–<5	102 (96%)	4 (4%)	98 (91%)	10 (9%)	0.26^a^	1.48 (0.74–2.95)
≥5–<10	155 (89%)	20 (11%)	143 (81%)	33 (19%)	0.16^a^	1.37 (0.79–2.35)
≥10	132 (78%)	37 (22%)	121 (67%)	59 (33%)	≤0.001	3.70 (2.3–5.95)

RAVI						
After 6 months up to 24 months	Lymphoedema defined by RAVI > 10%		Clinical sleeve application		*P*-value	Hazard Ratio
	No lymphoedema (*N* = 847)	Lymphoedema (*N* = 141)	No lymphoedema (*N* = 662)	Lymphoedema (*N* = 211)		
RAVI < 4%	633 (97%)	15 (3%)	540 (85%)	93 (14%)	NS	1
RAVI 4–9%	185 (72%)	71 (28%)	104 (56%)	81 (43%)	0.012	2.41 (1.21–4.80)
RAVI > 10%	39 (41%)	55 (59%)	18 (33%)	37 (67%)	≤0.001	3.09 (2.73–7.13)

*NS* non-significant.

^a^Non-significant: note RAVI changes predicted subsequent lymphoedema but BIS changes did not.

Sensitivity and specificity of BIS and RAVI were compared to the 221 patients with compression-sleeve application (clinical surrogate for lymphoedema) between 3 and 24 months. BIS identified high false positive numbers of patients with a ‘lymphoedema’ diagnosis whether the primary endpoint of RAVI > 10% or compression-sleeve application was used. Whilst the NPV for both BIS and RAVI > 10% were similar, the PPV was higher at all timepoints for RAVI > 10% because of the lower number of false positive results (between 6 and 24 months: (RAVI PPV 59%, 95% CI: 50–66% compared to BIS PPV 34%, 95% CI: 29–39%). Arm swelling was reported by 65·8% patients using the LBCQ^17^ by 24 months (Fig. 1).

### Quality-of-life

The minimum clinically important difference in Trial Outcome Index (TOI) is 5 points. Patients with lymphoedema at 6 (12, 18 and 24) months had lower FACT-B, TOI and ARM subscale scores (RAVI ≥ 10%: *p* = 0.027, *p* = 0.001, *p* < 0.001, respectively, Tables S1a, b and S2). At all timepoints, a higher percentage of patients reporting swelling or heaviness symptoms was found for those with lymphoedema (*p* < 0.001).

Multivariate analysis showed lymphoedema (by 12 months) *p* = 0.002, high BMI *p* < 0.001, and current smoking *p* = 0.036 all independently reduced TOI by 5 points (Table S2).

After compression-sleeve application (between 3 and 24 months), patient quality-of-life scores increased (Fig. S1b) (*p* < 0.001 for TOI, *p* = 0.001 for Total FACT-B, and ARM subscales Tables S3 and S4).^5^ The Estimated Marginal Mean (EMM) for TOI at baseline was 66.7 (95% CI: 64.7–68.6), 62·7 (95% CI: 60.6–64.7) before sleeve application and 66.8 (95% CI: 64.3–69.3) at 36 months. Quality-of-life following sleeve application increased for patients with a RAVI > 5% (*N* = 116), TOI increased from 62.4 (95% CI: 59.6–65.1) to 68.0 (95% CI: 65.2–70.7), whereas for those patients with RAVI < 5% (*N* = 86) TOI remained the same 65.3 (95% CI: 61.7–68.6) and 66.8 (95% CI: 63.1–70.1) Interaction: *p* = 0.022: Table S2).

### Predictors of Lymphoedema (RAVI>10%) from 1 month

The factors predicting lymphoedema diagnosis after ANC surgery from 1 to 36 months were assessed. Using multivariable logistic regression, RAVI at 1-month (*p* < 0.001), number of positive (not total removed) lymph nodes (*p* < 0.001), and prescription of chemotherapy (*p* = 0.008) were independent predictors of lymphoedema (from 1 to 36 months: Table 4).

**Table 4:** Prediction of Lymphoedema (defined by RAVI ≥ 10) by 36 months.

	Univariate		Multivariable		
Variable	OR (95% CI)	*p*-value	OR (95% CI)	*p*-value	Score
RAVI at 1 month		<0.001		<0.001	
<3%	1 (–)		1 (–)		0
≥3–<5%	1.37 (0.82–2.30)		1.48 (0.86–2.56)		0.5
≥5–<10%	4.08 (2.66–6.25)		5.27 (3.30–8.41)		1.5
>10%	5.54 (2.33–13.16)		6.60 (2.52–17.31)		2
LBCQ checklist swelling at pre-surgery (yes)	2.06 (1.22–3.49)	0.007	–	–	
Age (per year increase)	1.01 (1.00–1.03)	0.021	–	–	
BMI at pre-surgery		0.017		0.10	
≤25	1 (–)		1 (–)		0
>25–≤30	1.22 (0.83–1.79)		1.18 (0.75–1.86)		0
>30	1.72 (1.17–2.51)		1.63 (1.03 to +2.57)		0.5
ER status (negative)	1.32 (0.91–1.91)	0.14	1.35 (0.86–2.13)	0.19	0.5
Pos nodes(n) (per node inc)		<0.001		<0.001	
≤3	1 (–)		1 (–		0
4–9	1.28 (0.87–1.89)		1.41 (0.87–2.27)		0.5
≥10	2.77 (1.87–4.11)		3.05 (1.89–4.93)		1
Chemotherapy (CT)		0.015		0.008	
No CT	1 (–)		1 (–)		0.5
CT with no taxane	0.61 (0.30–1.25)		0.54 (0.23–1.24)		0
CT with taxane	1.41 (1.01–1.98)		1.57 (1.04–2.38)		1
Radiotherapy (RT)		0.015			
No RT	1 (–)		–	–	–
Local RT only	1.51 (0.94–2.43)				
Regional LN RT	1.99 (1.23–3.20)				
Stage		<0.001			
≤2	1 (–)		–	–	–
3	1.79 (1.32–2.42)				
B3 at pre-surgery		0.39			
0–1 (little to no swelling)	1 (–)		–	–	–
2–4 (some to considerable swelling)	1.28 (0.73–2.24)				

Prediction from 1 month thus allows lymphoedema risk to be assessed at the patients first postoperative clinic visit and counselling about risk given.

A scoring model, ranging from 0 to 5, was produced based on the regression coefficients of the multivariable logistic regression. Scores for RAVI (0–2), BMI (0–0.5), ER negativity (0–0.5), number of nodes involved (0–1) and chemotherapy (0–1) are added together to produce the score (Table 4). The area under the receiver operating curve (AUROC) of the scoring model was 0·71 (95% CI: 0.66–0.75). Using RAVI and the same factors at 6 months improved the AUROC marginally to 0.75 (5% CI 70–81%).

Out of 826 patients used for the model, 66% of patients had low scores (<1) at 1 month and 12% developed lymphoedema, whereas 30% who scored moderate risk (2–3) had a 32% risk of lymphoedema and 4% who scored high risk (score 3.5–4.5) had a 76.7% risk of lymphoedema by 36 months. Thus, using the model scores, potentially 66% of patients could be reassured regarding their low lymphoedema risk (11.6%) and resources concentrated on the moderate and high-risk groups for lymphoedema surveillance.

### Progression of Lymphoedema

At sleeve application 187 patients had a RAVI < 20% but despite compression-sleeves application for 22 (IQR 11–33) months, progression to RAVI ≥ 20% occurred in 29 cases (15.7%). Factors predicting progression to RAVI ≥ 20% after sleeve application (Table S5) were: RAVI > 10% at sleeve application, (for >10% vs <10% *p* = 0.025, OR = 3.08 (95% CI: 1.18–8.04), older age (*p* = 0.001, OR = 1.07, 95% CI: 1.03–1.12), BMI at application for >25 to ≤30 vs ≤25 (*p* = 0.015, OR 2·07, 95% CI: 0.62–11.1); for BMI > 30 vs ≤25: OR = 7.23, 95% CI: 1.74–29.9) and ER negativity (OR 3.70 95% CI: 1.26–10.86).

## Discussion

Increasing cancer survival will increase patients at risk of the consequences of their treatments. USA National Lymphoedema Network Guidelines^6^ recommend lymphoedema screening for all patients after axillary surgery or radiotherapy, which is frequently done using BIS alone. In this lymphoedema screening study, arm volume measurements more reliably diagnosed and predicted risk of lymphoedema, than BIS. The large cohort allowed accurate assessment of the diagnostic health technology involved but limits the strength inferring any causality about effects seen. Similar factors (RAVI > 5%, BMI > 30, and number of involved nodes) predicted development of lymphoedema, and its progression despite compression-sleeve therapy.

Screening for ‘subclinical’ lymphoedema is only appropriate if objective measures (RAVI > 5%) are used to allow early intervention with compression-sleeves and if this intervention reduces the risk of developing clinical lymphoedema.^16,17,19^

The lack of standardised diagnostic criteria for lymphoedema make estimation of the true incidence of lymphoedema difficult.^1,7–9^ Baseline measurements comparing both arms of individual women found 8.3% of patients exhibited marked arm volume (>200 ml) differences regardless of BMI, supporting the need for pre-surgery baseline measurements.^16,17^

Up to 25% of patients reported symptoms of swelling and/or heaviness^9^ in their ipsilateral limb before surgery. RAVI (not BIS) changes were associated with increased symptom reporting. Rigorous pre-operative baseline and subsequent measurements are required to determine the significance of any arm volume changes. Relative arm volume change is the optimal method for early diagnosis of lymphoedema.

DiSipio reviewed 30 prospective studies and estimated by 2 years around 21.4% patients developed lymphoedema after ANC.^1^

At the start of this study the BIS criteria for lymphoedema diagnosis was a 3sd increase but mainly on the basis of small cross-sectional studies a criterion of 2sd has been proposed.^20^

BIS (2sd or 3sd) assessed prospectively in a series with short follow-up time,^11^ whilst specific (85–92%), had a lower sensitivity and modest correlation with arm volume.^12,16,19^ Use of BIS measurements alone for early diagnosis would have led to threefold more patients having compression-sleeves applied inappropriately within 6 months (Table S2). A NICE review of limited data, found BIS was not as effective as current tests for diagnosing lymphoedema.^21^ Prospective BIS studies of sufficient size and length of follow-up of post ANC cancer patients have not been published but results from studies with 1-year follow-up support our longer-term findings.^12,16,19^

By 1 year, l-Dex increase of either 7.5 (51.8%) or 10 (39%) was identified in at least twice as many patients as RAVI > 10% (15.7%), a difference that remained constant at 60 months. The BIS criterion for a lymphoedema diagnosis at 6 months only identified 22% of women with lymphoedema (RAVI > 10%) by 24 months (Table 3). Supporters of BIS argue it identifies subclinical lymphoedema but with long term data it is clear it over diagnoses lymphoedema, as the rate of developing BIS > 7.5 or >10 was significantly higher at all timepoints than either RAVI > 10% or the clinical need for compression sleeves.

Arm Volume (RAVI) changes correlated with symptom development and quality-of-life, better than BIS.

Compared to either RAVI > 10% or sleeve application, BIS wrongly diagnosed (false positive) 12% patients with lymphoedema. Previous studies report, high false positives results occurred with BIS compared to Perometry.^12,16,19^ Use of BIS alone for screening for lymphoedema is therefore not recommended. However, defining lymphoedema as whole arm RAVI > 10% may underdiagnose those with segmental lymphoedema e.g. of hand or forearm. RAVI remains the preferred method and gold standard for diagnosis and treatment.

If Perometry is unavailable, tape measurements are an alternative to calculate volume changes.

Self-reported symptoms often lead to lymphoedema treatment.^4,8,22^ A reliance on symptoms alone to diagnose lymphoedema is inappropriate as 8% have symptoms before surgery, 43% of patients reported symptoms of swelling and/or heaviness in their limb by 6 months potentially resulting in over diagnosis and treatment indicating the importance of consistent, objective and robust measurement techniques.

The Perometer is the most objective tool to measure arm swelling. Treatment decisions to apply compression sleeves based on subjective information (patient reported arm symptoms) have led to lymphoedema staging classifications describing a prodromal or latent phase of lymphoedema characterised by arm heaviness or swelling. This study suggests that application of a compression sleeve based upon clinical symptoms of swelling and/or heaviness alone in the absence of a measurable change in RAVI is likely to lead to misdiagnosis and overtreatment. In the absence of a RAVI > 5%, quality-of-life was not improved by sleeve application.

Screening for ‘subclinical’ lymphoedema to enable intervention to reduce the risk of developing clinical lymphoedema is recommended internationally,^10,17^ despite the weak evidence base. For ‘subclinical’ lymphoedema, prescribing of ‘prophylactic’ compression-sleeves has occurred internationally without any randomised evidence of efficacy. Nearly 16% of our women who had treatment compression-sleeves (20–25 mmHg) applied progressed to moderate lymphoedema despite claims that intervention with compression sleeves prevented lymphoedema development. Other studies report 35% of lymphoedema patients progress after sleeve application.^23^ A randomised controlled trial of External Compression Sleeves (20–25 mmHg) Therapy + ‘standard management’ (written advice, arm elevation, exercises and massage) versus ‘standard management’ alone to prevent LE found no difference in LE rate between the interventions.^24^

Screening can be used with the aim of detecting subclinical lymphoedema to allow early intervention to try to reduce the risk of clinical lymphoedema developing. In addition, screening can be used with the aim of detecting early clinical lymphoedema so that early intervention can be introduced to reduce the risks of progression of the swelling and of the development of the main complication of cellulitis. Anecdotal clinical experience internationally confirms that intervention with compression garments in early lymphoedema reduces the risk of the development of more severe swelling. However, as stated above, evidence that early intervention in subclinical lymphoedema reduces the risk of developing lymphoedema is lacking.^24^

Increasingly cancer patients are encouraged to improve their survivorship by self-management. Our approach was to combine factors to produce a novel scoring index to allow identification of patients at risk of the condition who may benefit from lymphoedema surveillance. Early arm swelling after surgery (RAVI at 1 month or indeed at 6 months) was a good predictor of subsequent lymphoedema and the scores were weighted for each factor according to the risk of subsequent lymphoedema. This index, if validated, would allow clinicians to give extra attention to support at risk patients following the post-operative visit.

One of the challenges with this approach is that even in the low risk group, there is an 11% chance of developing lymphoedema by 36 months and, therefore, the risk cannot be ignored altogether. A possible solution is that patients in the low risk group can be informed of signs and symptoms of lymphoedema, given written advice, taught simple lymphatic massage, encouraged to be vigilant about self-screening using symptoms or signs and to self-refer to a lymphoedema clinic if they develop any new symptoms.

Alternatively, if resources permit, continued surveillance of this group could take place. However, patients may find continued attendance at the lymphoedema clinic at 3 or 6 monthly intervals, anxiety provoking. In our study many failed to attend beyond 2 years, as they were attending a separate annual cancer clinic.

We feel that within the lymphoedema screening debate whether screening is for reducing the risk of developing lymphoedema or of the progression to and complications of more severe lymphoedema, delayed treatment of established lymphoedema is no longer acceptable, but neither is a paternalistic approach which fails to address anxiety and explain individualised risk to patients. Our model allows reassurance, explanation of individualised risk and the potential discharge from regular surveillance of those who score “low risk” in the model. Nevertheless, those at low risk should still be given advice and guidance about reducing the risk of developing lymphoedema and how to recognise it early should it develop.

Sentinel node biopsy (SNB), causes lymphoedema in 5–15% patients and development of lymphoedema after SNB is predicted by arm volume (RAVI) changes.^2,25^ After ANC, early arm swelling (RAVI > 5%) post-surgery was a strong predictor of lymphoedema, as were BMI and number of involved lymph nodes.

Conflicting evidence exists as to whether weight gain after breast cancer surgery or BMI alone is a predictor of lymphoedema^1,25^ or not. Baseline BMI > 30 predicted lymphoedema in a large RCT of sentinel node biopsy.^20^ A systematic review found that overall increases in BMI elevated lymphoedema incidence.^1^ In our study, BMI at surgery was an independent predictor of both reduced QoL and risk of lymphoedema after ANC, but the main effect of BMI was on risk and progression of lymphoedema after sleeve application. Although interventions to reduce weight after cancer diagnosis are effective, substantial change in BMI after surgery was rare. This is particularly true in patients with BMI > 30 in whom less than 5% reduced BMI by 5 units. Interventions to lower BMI may reduce lymphoedema occurrence and improve quality-of-life.^26^ The increases in obesity (BMI) internationally in developed nations is likely to increase cancer incidence, the burden of lymphoedema and relapse after treatment. Greater attention to educating public and patients about healthy weight management is required, and future approaches must include behaviour change techniques.^26^

## Conclusions

Preoperative objective arm volume measurements (rather than BIS), are essential to the appropriate management of lymphedema after breast cancer treatment. Such objectivity would reduce NHS costs and ensure that patients who will benefit from compression therapy are offered treatment. Self-reported symptoms are common after surgery and need corroboration with arm volume measurements.

Prescription of compression sleeves in lymphoedema, improves symptoms and quality-of-life. High BMI increases development and progression of lymphoedema. Weight reduction strategies are required to prevent lymphoedema developing after axillary surgery and radiotherapy.

## Supplementary information

Revised supplementary material

## Data Availability

The data and material is all available through writing to Manchester CTU (formerly MAHSCCTU).
