# Supplementary material for: Increases in arm volume predict lymphoedema and quality of life deficits after axillary surgery: a prospective cohort study
Source: Br J Cancer. 2020 May 4;123(1):17–25. doi: 10.1038/s41416-020-0844-4 (PMC7341763; doi:10.1038/s41416-020-0844-4)
Supplement: Supplementary file 1 — Revised supplementary material [file 41416_2020_844_MOESM1_ESM.docx]

**Supplementary material**

**Legends to Figures and Tables**

**Figure S1A: The changes in FACT-B TOI scores over time post-surgery by sleeve application (*N*=1002)** (means and 95% Confidence Intervals presented in diagrams)

**S1B:** **Effect of Sleeve Application on Quality of Life (TOI and FACT-B total scores) (*N*=214)** (means and 95% Confidence Intervals presented in diagrams)

**Table S1: Change in absolute RAVI and BIS from 6 to 24 months compared to lymphoedema checklist symptoms at 24 months**

**Table S2: Factors affecting Trial Outcome Index (Quality of Life)**

TOI reduction of greater than 5 is clinically significant and apparent in Lymphoedema diagnosis, BMI and cigarette smokers. Although statistically significant the other variables were not clinically significant.

**Table S3: QoL benefit following sleeve application in subgroups of patients with RAVI ≥5% or patient reporting B3 2-4 (considerable swelling) and patients with RAVI <5% and B3 0-1 (little to no swelling)**

RAVI>5% patients with arm swelling experienced improved QoL scores but patients with RAVI<5% on sleeve application did not improve QoL

**Table S4: QoL values in those with and without lymphoedema at 6, 12, 18 and 24 months**

**Table S5: Factors predicting progression to Moderate (RAVI>20%) lymphoedema despite sleeve application**

**BEA / PLACE Trial Protocol**

**Supplementary material**

**Figure S1A: The changes in FACT-B TOI scores over time post-surgery by sleeve application (*N*=1002)** (means and 95% Confidence Intervals presented in diagrams)

**S1B:** **Effect of Sleeve Application on Quality of Life (TOI and FACT-B total scores) (*N*=214)** (means and 95% Confidence Intervals presented in diagrams)


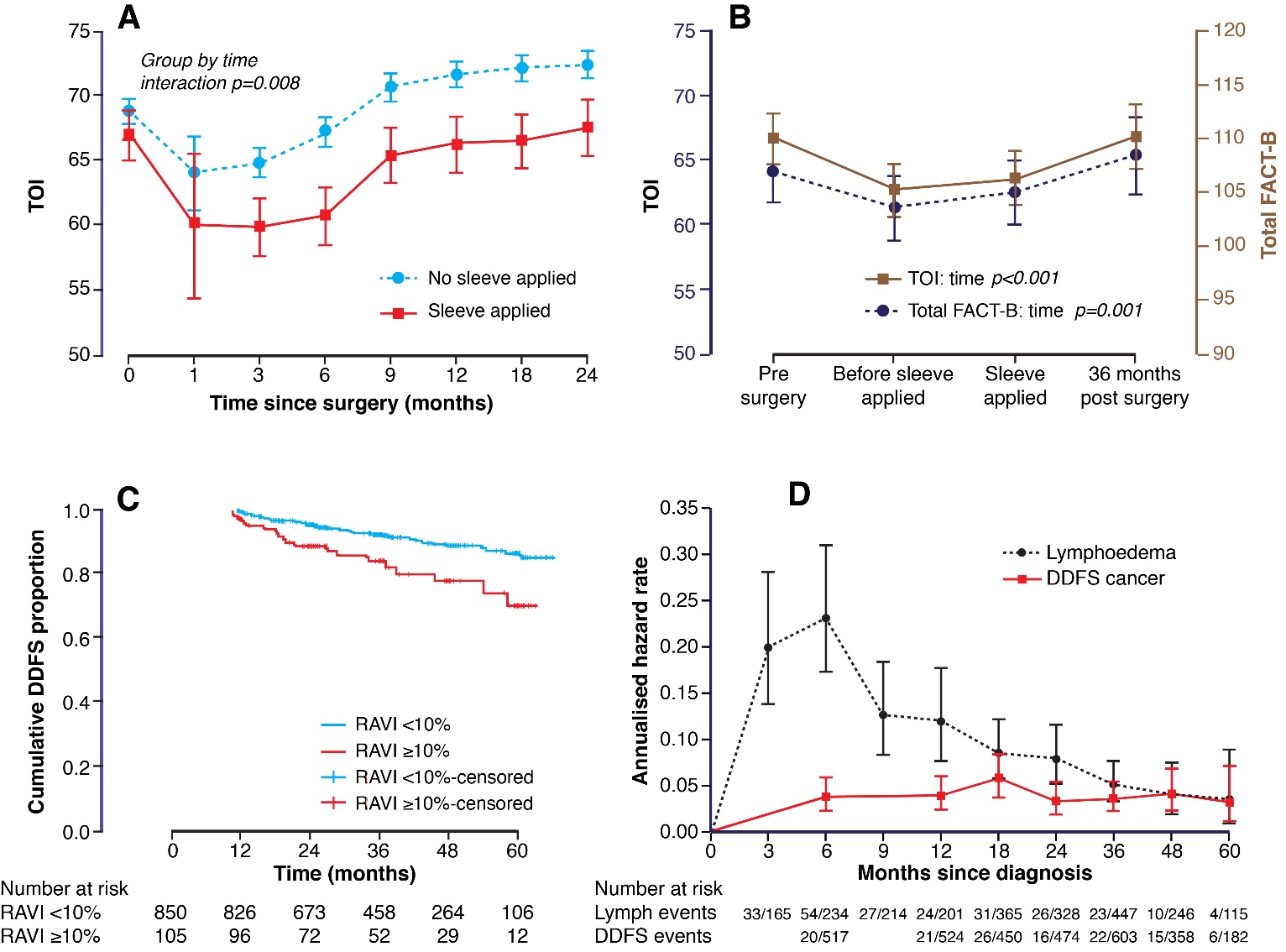


**Table S1: Change in absolute RAVI and BIS from 6 to 24 months compared to lymphoedema checklist symptoms at 24 months**

| Change from 6 months to 24 months  Mean (SD) | No swelling at 24 months | Swelling at 24 months | *P*-value |
| --- | --- | --- | --- |
| RAVI | *N*=323  -0.4 (4.7) | *N*=222  2.0 (8.0) | <0.001 |
| BIS | *N*=310  -0.3 (8.0) | *N*=206  -1.0 (12.7) | 0.48 |
|  | No numbness at 24 months | Numbness at 24 months |  |
| RAVI | *N*=145  0.1 (5.7) | *N*=408  0.8 (6.6) | 0.28 |
| BIS | *N*=139  -1.8 (9.4) | *N*=385  -0.2 (10.2) | 0.12 |
|  | No heaviness at 24 months | Heaviness at 24 months |  |
| RAVI | *N*=303  -0.2 (5.3) | *N*=231  1.7 (7.5) | 0.001 |
| BIS | *N*=290  -0.8 (8.4) | *N*=216  -0.4 (11.9) | 0.69 |

**Table S2: Trial Outcome Index (TOI) analysis**

|  |  |  | Univariate | |  | Multivariable (*N*=684) | |
| --- | --- | --- | --- | --- | --- | --- | --- |
| Variable |  | *N* | EMM (95% CI) | *P*-value | *N* | EMM (95% CI) | *P*-value |
| Lymphoedema(RAVI>10% by 12 months | No | 613 | 72.3 (71.3-73.4) | 0.001 | 593 | 69.4 (67.4-71.3) | 0.002 |
|  | Yes | 96 | 67.1 (64.0-70.1) |  | 91 | 64.3 (60.2-67.9) |  |
| Chemotherapy | No | 224 | 73.3 (71.6-74.9) | 0.10 |  | - | - |
|  | Adjuvant | 443 | 71.0 (69.7-72.3) |  |  | - |  |
|  | Neo | 37 | 71.4 (66.8-75.5) |  |  | - |  |
| Type of Chemotherapy | No | 224 | 73.3 (71.6-74.9) | 0.020 | 215 | 69.3 (66.7-71.8) | 0.055 |
|  | No taxane | 45 | 67.4 (62.7-71.5) |  | 43 | 63.7 (58.2-68.6) |  |
|  | Taxane | 436 | 71.4 (70.1-72.6) |  | 426 | 67.4 (65.2-69.6) |  |
| Right Lymph Node RT | No RT | 130 | 72.1 (69.7-74.3) | 0.018 | 125 | 66.5 (63.0-69.7) | 0.029 |
|  | No RLNR | 317 | 73.0 (71.6-74.4) |  | 311 | 68.7 (66.0-71.2) |  |
|  | RLNR | 261 | 69.8 (68.1-71.5) |  | 248 | 65.5 (62.4-68.3) |  |
| Stage | ≤2 | 434 | 72.5 (71.2-73.7) | 0.031 |  | - | - |
|  | 3 | 272 | 70.2 (68.5-71.9) |  |  | - |  |
| BMI at baseline | ≤25 | 249 | 75.2 (73.7-76.6) | <0.001 | 244 | 71.3 (68.6-73.7) | <0.001 |
|  | >25-≤30 | 262 | 70.9 (69.3-72.5) |  | 258 | 66.1 (63.1-69.0) |  |
|  | >30 | 187 | 67.3 (65.2-69.4) |  | 182 | 62.8 (59.4-66.0) |  |
| Type of surgery | ANC/Other | 187 | 73.5 (71.6-75.3) | 0.021 |  | - | - |
|  | WLE+ANC | 205 | 72.2 (70.3-74.0) |  |  | - |  |
|  | Mast+ANC | 320 | 70.2 (68.6-71.7) |  |  | - |  |
| Smoking | Never | 432 | 72.6 (71.4-73.8) | 0.006 | 417 | 69.1 (66.7-71.2) | 0.036 |
|  | Ex-smoker | 220 | 71.2 (69.3-72.9) |  | 211 | 67.7 (64.9-70.3) |  |
|  | Current | 59 | 66.4 (62.3-70.2) |  | 56 | 63.7 (58.8-68.1) |  |
| Age | <50 | 232 | 70.9 (69.1-72.7) | 0.32 | 222 | 65.7 (62.4-68.7) | 0.047 |
|  | ≥50 | 480 | 72.0 (70.8-73.2) |  | 462 | 68.1 (65.7-70.4) |  |

RT = Radiotherapy; BMI = Body Mass Index; Mast = Mastectomy; ANC = Axillary Node Clearance; WLE = Wide Local Excision

**Table S3: QoL benefit following sleeve application in subgroups of patients with RAVI ≥5% or patient reported B3 2-4 (considerable swelling) and patients with RAVI <5% and B3 0-1 (little to no swelling)**

|  |  |  | **Estimated marginal mean**  **(95% confidence intervals)** | | | ***P* value** |
| --- | --- | --- | --- | --- | --- | --- |
|  |  | **N** | **At sleeve application** | **Approximately 6-12 months after sleeve was applied** | **At 48 months post surgery** |  |
| **FACT- B+4 total^1^** | **RAVI<5 and B3 score of 0-1** | 40 | 112.8  (106.5-118.4) | 112.7  (106.0-118.7) | 106.3  (98.2-113.5) | Time: 0.12  B3 score: 0.28  Interaction: 0.001 |
|  | **RAVI ≥5 or B3 score of 2-4** | 153 | 102.5  (99.0-105.9) | 107.5  (103.5-111.2) | 110.1  (105.4-114.4) |  |
| **TOI^2^** | **RAVI<5 and B3 score of 0-1** | 40 | 70.9  (67.0-74.5) | 71.1  (66.6-75.1) | 66.6  (60.7-71.8) | Time: 0.042  B3 score: 0.063  Interaction: <0.001 |
|  | **RAVI ≥5 or B3 score of 2-4** | 153 | 61.3  (58.7-63.7) | 65.9  (63.3-68.5) | 68.0  (64.7-71.1) |  |
| **ARM^3^** | **RAVI<5 and B3 score of 0-1** | 40 | 15.7  (14.8-16.5) | 14.9  (13.8-15.9) | 14.7  (13.4-15.8) | Time: 0.26  B3 score: 0.33 Interaction:  <0.001 |
|  | **RAVI≥5 or B3 score of 2-4** | 154 | 13.4  (12.7-14.1) | 14.5  (13.8-15.1) | 15.7  (14.8-16.6) |  |

^1^Analysis was performed using LN(170-FACT-B)

^2^Analysis was performed using LN(110-TOI)

^3^Analysis was performed using LN(23-ARM)

**Table S4: QoL values in those with and without lymphoedema at 6, 12, 18 and 24 months**

|  | FACT-B | |  |
| --- | --- | --- | --- |
| Lymphoedema at… | Median, IQR (range) | | *P*-value |
| Time (n=no: n=yes) | Lymphoedema -No | Lymphoedema -Yes |  |
| Baseline | 110.9 (109.7-112.2) | 109.4 (105.0-113.4) | 0.48 |
| 6 months (660:58) | 110, 95-124 (21-144) | 104, 87-114 (42-138) | 0.027 |
| 12 months (628:55) | 117, 101-129 (34-144) | 104, 89-120 (28-141) | 0.004 |
| 18 months (566:59) | 118, 102-129 (33-144) | 109, 96-121 (49-141) | 0.005 |
| 24 months (541:68) | 118, 102-130 (31-144) | 113, 95-128 (31-144) | 0.085 |
|  | | | |
|  | Trial Outcome Index (TOI) | |  |
| Lymphoedema at… | Median, IQR (range) | |  |
| Time (n=no: n=yes) | Lymphoedema -No | Lymphoedema -Yes |  |
| Baseline | 68.3 (67.4-69.2) | 67.0 (63.9-70.0) | 0.42 |
| 6 months (669:59) | 67, 55-77 (5-92) | 60, 50-66 (20-88) | 0.001 |
| 12 months (637:56) | 73, 60-80 (9-92) | 64, 54-75 (9-90) | 0.001 |
| 18 months (570:63) | 73, 62-81 (16-92) | 67, 59-76 (27-89) | 0.001 |
| 24 months (546:70) | 74, 64-82 (17-92) | 69, 57-78 (11-92) | 0.009 |
|  | | | |
|  | ARM subscale | |  |
| Lymphoedema at… | Median, IQR (range) | |  |
| Time (n=no: n=yes) | Lymphoedema -No | Lymphoedema -Yes |  |
| Baseline | 18.7 (18.6-18.9) | 18.6 (17.9-19.1) | 0.62 |
| 6 months (688:60) | 16, 13-18 (0-20) | 14, 10-16 (0-20) | <0.001 |
| 12 months (654:56) | 16, 14-18 (0-20) | 14, 10-17 (0-20) | <0.001 |
| 18 months (583:64) | 16, 14-18 (0-20) | 14, 10-17 (0-20) | <0.001 |
| 24 months (558:74) | 17, 14-19 (0-20) | 15, 10-17 (0-20) | <0.001 |

Median (Interquartile Ranges:IQR).

**Table S5: Factors predicting progression to Moderate (RAVI>20%) lymphoedema despite sleeve application**

|  |  | **Univariate** | | | **Multivariable** | |
| --- | --- | --- | --- | --- | --- | --- |
| Variable | *N* (with RAVI ≥20%) | OR (95% CI) | *P*-value | *N* (with RAVI ≥20%) | OR (95% CI) | *P*-value |
| **RAVI at sleeve application**  <5%  ≥5-<10%  ≥10-<20% | 83 (9)  58 (8)  44 (12) | 1 (-)  1.32 (0.48-3.64)  3.08 (1.18-8.04) | 0.056 | - | - | - |
| **ARM subscale at sleeve** application (per increase in score) | 161 (24) | 1.02 (0.91-1.14) | 0.74 | - | - | - |
| **Lymphoedema Checklist Swelling** at sleeve application  No  Yes | 12 (0)  141 (23) | -  - | 0.21* | - | - | - |
| **B3 at sleeve application**  0-1 (little to no swelling)  2-4 (considerable swelling) | 74 (7)  87 (17) | 1 (-)  2.32 (0.91-5.96) | 0.079 | - | - | - |
| **Age** (per year increase) | 185 (29) | 1.06 (1.02-1.10) | 0.001 | 168 (25) | 1.07 (1.03-1.12) | 0.001 |
| **BMI at sleeve application**  ≤25  >25-≤30  >30 | 56 (3)  62 (10)  54 (13) | 1 (-)  3.40 (0.88-13.05)  5.60 (1.50-20.97) | 0.037 | 55 (3)  59 (9)  54 (13) | 1 (-)  2.63 (0.62-11.11)  7.23 (1.74-29.99) | 0.015 |
| **ER status**  Positive  Negative | 151 (20)  30 (8) | 1 (-)  2.38 (0.93-6.07) | 0.069 | 140 (17)  28 (8) | 1 (-)  3.70 (1.26-10.86) | 0.017 |
| **Number Nodes positive**  ≤3  4-9  ≥10 | 117 (16)  39 (5)  29 (8) | 1 (-)  0.93 (0.32-2.72)  2.40 (0.91-6.34) | 0.17 | - | - | - |

*Fisher’s exact test used because no patients with RAVI ≥20% in the ‘no swelling’ category

BMI Body Mass Index, FACT B+4 B3 question arm swelling scored 0-4

**BEA / PLACE Trial Protocol**

The BEA study comparing bioimpedance with perometer measurement was a prospective study to identify patients suitable for including in a randomised trial comparing compression sleeves with control management for one year. The PLACE trial will be reported separately.

As the protocol indicated, the primary end point was Lymphoedema defined by Relative Arm Volume Increase (RAVI) >10%, as this was the standard in the United Kingdom.

All data was collected on clinical report forms sent to the MAHSC-Clinical Trials Unit in a prospective manner. The protocol for the PLACE trial is online: <http://www.isrctn.com/ISRCTN92355292>. It was anticipated before the trial was started there were multiple potential criteria measures of lymphoedema, but RAVI>10% estimated by the following formula was the primary endpoint:

$$\left( \frac{A2-U2}{U2} \right)X100- \left( \frac{A1-U1}{U1} \right)X100$$

Where the volume of the treated arm (A2) minus the baseline volume of the treated arm over the baseline volume of the treated arm multiplied by 100 and minus the contralateral arm (A1) minus the original arm baseline arm volume (U1) over the original arm baseline arm volume multiplied by 100.

Perometer measurements were undertaken twice and the data averaged. Each perometry measurements had to be within one standard deviation or else a third measurement was taken by an independent nurse and the data averaged. The protocols were in place at the commencement of the trial and arm volume was determined using standard perometer measurements. The perometer determines arm volume data from the shoulder to the wrist.

Calculations of volume increase were carried out from baseline, which eliminated issues of dominant arm, non-dominant arm volume. The nature of the calculation of absolute volume difference depends on the baseline values and most patients developed an absolute volume difference over 24 months of at least 100ml. Indeed, by 24 months, 25.5% of patients had a volume greater than 200ml and the difference was less when the dominant arm was compared to the non-dominant arm (119 out of 717 patients had a dominant arm - non-dominant arm increase of >200ml, compared to dominant arm - non-dominant arm >100ml was 34.7% (249 out of 717 patients in 24 months)).

The table below shows the arm volume change over time for dominant and non-dominant arms and indicates that the increase of median arm volume increases greater than 100ml was similar in both the dominant and the non-dominant arm. This data relates to all patients in the study and does not relate purely to the lymphoedema patients. The details of the quality of life are given in the manuscript methodology, currently page 8 and reference 5 (Coster S, Poole K, Fallowfield LJ The validation of a quality of life scale to assess the impact of arm morbidity in breast cancer patients post-operatively. Breast Cancer Res Treat (2001) 68: 273. <https://doi.org/10.1023/A:1012278023233>).

| Timepoint | n | Median (range) for dominant arm | Dominant arm from baseline ≥ 200mL | n | Median (range) for non-dominant arm | Non-dominant arm from baseline ≥ 200mL |
| --- | --- | --- | --- | --- | --- | --- |
| 1 month | 852 | -44.5 (-1770-1164) | 67 (7.9%) | 852 | -41.0 (-1635-812) | 69 (8.1%) |
| 3 months | 877 | 8.0 (-1401-1357) | 128 (14.6%) | 876 | 21.0 (-1437-1371) | 128 (14.6%) |
| 6 months | 852 | 29.5 (-1181-2377) | 189 (22.2%) | 851 | 39.0 (-1303-2471) | 199 (23.4%) |
| 9 months | 700 | 31.0 (-1109-2230) | 152 (21.7%) | 700 | 35.0 (-947-2215) | 157 (22.4%) |
| 12 months | 824 | 35.5 (-1419-2317) | 197 (23.9%) | 823 | 34.0 (-1312-2249) | 206 (25.0%) |
| 18 months | 741 | 47.0 (-1515-2349) | 185 (25.0%) | 740 | 55.0 (-1289-2296) | 212 (28.6%) |
| 24 months | 716 | 53.5 (-1709-1913) | 199 (27.8%) | 715 | 66.0 (-1484-1997) | 203 (28.4%) |

n = number of patients with data at each timepoint.

There is no statistically significant difference between the dominant arm and the non-dominant arm volume change at any of the timepoints using a Wilcoxon signed-rank test.

**BIS**

Training for BIS was provided by the ImpediMed company (Carslbad, CA, USA; [www.impedimed.com](http://www.impedimed.com)), with a company representative sent to the UK to provide training for all the centres. In addition, a CD-ROM of the L-Dex methodology and the training was provided to each of the centres, prepared and approved by ImpediMed. The patients were measured in a supine position on a bed. L-Dex readings were checked prior to acceptance and the devices were tested regularly by ImpediMed and the centres themselves. Placement of electrodes was as advised by ImpediMed and as illustrated in the CD-ROM.

**Compression garments**

Compression knitted off the shelf garments were provided by Sigvaris to the trial without charge. The compression was from 20mgHg at the wrist to 25mgHg at the shoulder. Women were assessed without compression across the additional time points, having been asked to take their compression garment off before measurement. Compliance with compression was good and was assessed as part of the study.

**Quality of Life Questionnaires**

A Lymphoedema Breast Cancer Questionnaire which asks three questions on heaviness, numbness and swelling and the presence or absence of those symptoms was given to all patients. In addition, a functional assessment of cancer therapy, breast plus forearm mobility, quality of life questionnaire FACT-B+4, which has been validated in several breast cancer trials was used. The Trial Outcome Index (TOI) was the primary outcome as reductions of TOI of five or more units are clinically relevant. The FACT-B+4 provides a number of summary scores, including physical wellbeing, functional wellbeing and the breast cancer subscale.

In addition, the four questions in a scale of 1-5 for arm swelling, numbness, shoulder movement, arm movement and arm heaviness are part of the FACT-B+4 scoring system. Data on heaviness and swelling and their presence according to the FACT-B+4 linear analog scale calculation of the presence of these symptoms is presented in Figure 1. Thus if these symptoms were present, they were also on the linear analog scale of 1-4, whereas if the symptoms were not present a score of 0 was on the linear analog scale.
